# Genomic Characterizations of a Newcastle Disease Virus Isolated from Ducks in Live Bird Markets in China

**DOI:** 10.1371/journal.pone.0158771

**Published:** 2016-07-08

**Authors:** Jingjing Wang, Yan Lv, Yi Zhang, Dongxia Zheng, Yunling Zhao, David Castellan, Hualei Liu, Zhiliang Wang

**Affiliations:** 1 OIE Reference Laboratory for Newcastle Disease, China Animal Health and Epidemiology Center, Qingdao 266032, China; 2 OIE Collaborating Centre for Veterinary Epidemiology and Public Health, China Animal Health and Epidemiology Center, Qingdao 266032, China; 3 DM Castellan International Veterinary Consulting, Niagara Falls, Canada; Oklahoma State University, UNITED STATES

## Abstract

One class I Newcastle disease virus (NDV), designated as duck/Guangxi/1261/2015 (GX1261), was isolated from asymptomatic ducks in live bird markets (LBM) from southern China during the national active surveillance for NDVs in 2015. The complete genome length of GX1261 isolate was 15,198 nucleotides with the gene order of 3’-NP-P-M-F-HN-L-5’. The motif at the cleavage site of F protein was ^112^ERQER/L^117^, which was typical of low virulence NDV. Several mutations were identified in the functional domains of F and HN proteins, including fusion peptide, heptad repeat region, transmembrane domains and neutralizing epitopes. Phylogenetic analysis based on the complete F gene revealed that the isolate was clustered into sub-genotype 1c in class I, and showed a high level of similarity with the strains isolated from waterfowl in the United States of America. This is the first report of this kind of virus in the mainland of China. These results demonstrated that GX1261-like viruses might exist in asymptomatic waterfowl, and remain undetected or unidentified. Thus, more investigation needs to be done in order to identify the source of the virus. This study revealed the genetic and phylogenetic characteristics of GX1261 isolate and could help us to better understand the epidemiological context of class I NDV in China.

## Introduction

Newcastle disease virus (NDV), also termed as avian paramyxovirus type 1 (APMV-1), belongs to the genus *Avulavirus* in the family *Paramyxoviridae*. The virulent strains of NDVs with the intracerebral pathogenicity index (ICPI) ≥ 0.7 are the cause of Newcastle disease, which is one of the most serious infectious diseases in poultry industry and must be reported to the World Organization for Animal Health (OIE) [1,2,3]. NDV has a negative sense, single stranded RNA genome with at least three sizes: 15,186, 15,192 and 15,198 nucleotides (nt), and contains six genes coding for the nucleocaspid protein (NP), phosphoprotein (P), matrix protein (M), fusion protein (F), haemagglutinin-neuraminidase (HN), and a large polymerase protein (L) [4,5]. Pathotypically, NDVs can be classified into 3 groups, lentogenic, mesogenic and velogenic strains, based on virulence phenotype [6]. The velogenic and mesogenic strains are currently defined as virulent NDVs, while lentogens are viruses with low virulence [1]. Genetically, NDVs have been divided into two classes, namely class I and class II. Class II strains have been isolated from wild and domestic birds, including virulent and low-virulent stains, and contain at least 18 genotypes [7]. Class I strains have mainly been isolated from wild birds and are low-virulent. In the old classification system, class I strains were divided into at least 9 genotypes [1], while in the new classification system, they have been condensed into a single genotype with at least 3 sub-genotypes [8].

Live bird markets (LBMs) are considered to be a source of respiratory pathogens, such as avian influenza virus (AIV) and NDV, which cause disease in poultry [9,10,11]. In China, both class I and class II NDVs with different genotypes and sub-genotypes have been isolated from LBMs widely [12,13,14]. Some studies have suggested that waterfowl could harbor lentogenic NDV strains and act as a natural reservoir for NDV, which plays an important role in viral evolution [1,15,16,17]. Moreover, some lentogenic strains have the potential to become virulent through transmission or circulation in poultry populations [18]. Thus, it is necessary to study the viruses isolated from waterfowl to better understand the viral evolution and genetic characteristics, and bring the early warning on emergence of novel NDVs. In this study, one class I NDV obtained from a duck in the LBM of southern China during the active surveillance in 2015 was sequenced and analyzed. Our results revealed the genetic and phylogenetic characteristics of this isolate and showed amino acid mutations in functional domains of F and HN proteins.

## Materials and Methods

### Ethics statement

This study was conducted according to the guidelines of animal welfare of World Organization for Animal Health, and approved by the Animal Welfare Committee of China Animal Health and Epidemiology Center (Permit number: 2015-CAHECAW-08). Swabs collected from the poultry in LBMs were approved by the owners of LBMs.

### Virus isolation and identification

**Fig 1:**
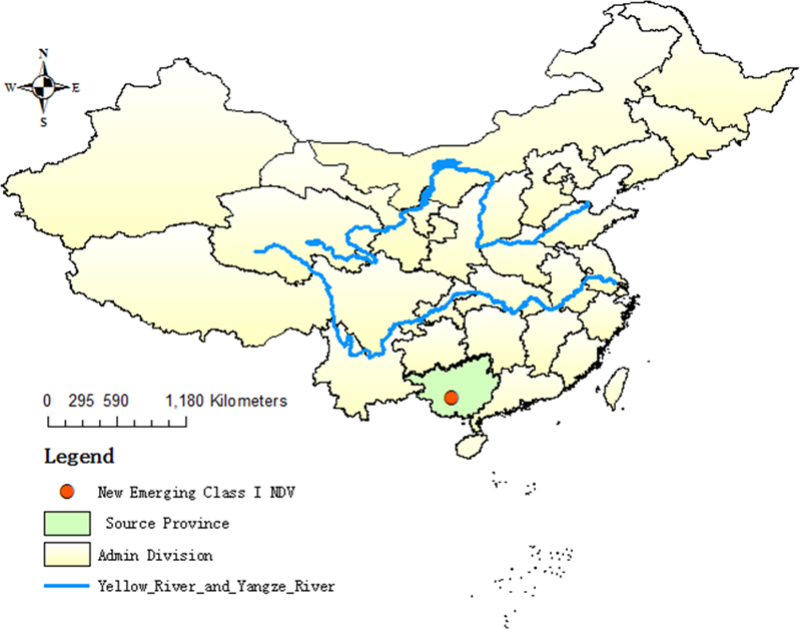
LBM where class I NDV (GX1261) was isolated.

Tracheal as well as cloacal or fecal swabs were collected randomly from 185 asymptomatic ducks in one LBM (22°49′60″N, 108°18′11″E) in Nanning city of Guangxi province in China (Fig 1) during the active surveillance program in 2015. The ages of ducks range from 50 to 100-day-old. All samples were obtained by our group and propagated in 9 to 11-day-old specific-pathogen-free (SPF) eggs for 72 h. The allantoic fluid was collected and identified by standard hemagglutination (HA) assay and reverse transcription polymerase chain reaction (RT-PCR). The isolate GX1261 was plaque purified through 3 passages on chicken fibroblast (DF1) cells.

### RNA extraction, RT-PCR and sequencing

The RNA extraction and RT-PCR were conducted as described previously [13]. Viral genomic RNA was extracted using High Pure Viral RNA Kit (Roche Applied Science) and amplified by RT-PCR with SuperScript III One-Step RT-PCR Platinum Taq HiFi (Invitrogen). Ten pairs of overlapping specific RT-PCR primers were designed based on the available NDV sequences from GenBank. For the 3’ leader and 5’trailer, primers were designed based on the specific sequence of duck/Guangxi/1261/2015, and the RNA was amplified using 3’-Full RACE Core Set Ver.2.0 (Takara) and 5’-Full RACE Kit (Takara) respectively according to the manufacturer’s instructions. Primer sequences used to amplify the genome are shown in S1 and S2 Tables. The amplified products were sequenced at Beijing Genomics Institute, China.

### Sequence analysis and phylogenetic studies

The nucleotide sequence assembly, editing, prediction of amino acid sequences, alignments, and analysis of the complete genome and the F, HN proteins were conducted with the Lasergene sequence analysis software package (DNAStar) and MEGA. Sequences of vaccine strains (La Sota and V4) and 12 representative NDV strains were downloaded from GenBank. The consensus amino acid sequence was derived from NDV strains of different genotypes as described previously [19]. For phylogenetic analysis, the sequences of the complete genome or F gene open reading frame (ORF) of GX1261 and other representative strains were aligned using the Clustal W multiple alignment algorithm in MEGA. The phylogenetic tree based on complete genomes or F gene ORFs was constructed by MEGA using the neighbor-joining method with 1000 bootstrap replicates. The sequences used for phylogenetic analysis were downloaded from GenBank, and the GenBank accession numbers are shown in phylogenetic trees.

## Results

### Virus isolation and identification

The NDV isolate was identified by HA and RT-PCR assays and designated as duck/Guangxi/1261/2015 (GX1261). The LBM (22°49′60″N, 108°18′11″E) where GX1261was isolated is shown in Fig 1.

### Genomic characteristics

Sequences of ten overlapping fragments and of 5’ and 3’ ends, covering the whole genome, were obtained by RT-PCR and assembled into one contiguous sequence. The full-length sequence of GX1261 consisted of 15,198 nt, which followed the “rule of six” and the order 3’-NP-P-M-F-HN-L-5’, and had the highest identity (97.6%) with the sequence of strain Goose/Alaska/415/91 (GenBank accession number AB524405). When compared with the genomic sequence (15,186 nt) of the La Sota strain, GX1261 had a 12 nt insertion (TGGGAGACAGGG) in the coding region of P gene between nucleotides 2,381 and 2,382, which is similar to that of the class I strain duck/China/JS10/2010 (JS10) [20]. The nucleotide sequence identity of the complete GX1261 genome and six genes to those of 12 other representative NDV strains is shown in Table 1.

**Table 1:** The nucleotide sequence identity of GX1261 and other representative NDV strains.

Strains	GenBank accession numbers	Class-genotype	Nucleotide sequence identity (%)								
			Complete genome	Leader	NP	P	M	F	HN	L	Trailer
GX1261	KU748779	I-1c	100	100	100	100	100	100	100	100	100
Goose/Alaska/415/91	AB524405	I-1c	97.6	96.4	96.8	97.3	97.9	97.0	97.5	98.1	98.3
JS10	HQ008337	I-1c	96.3	96.4	95.7	95.6	96.2	95.2	96.2	96.9	98.3
NDV08-004	FJ794269	I-1a	93.2	94.6	92.5	91.5	92.8	93.4	92.2	94.2	90.4
V4	JX524203	II-I	73.2	87.5	75.3	65.3	76.7	71.6	70.0	75.0	57.4
La Sota	KC844235	II-II	72.3	85.7	73.4	66.6	75.2	69.8	69.0	74.4	58.3
Mukteswar	JF950509	II-III	72.9	87.5	73.8	65.1	74.4	70.8	69.5	75.4	58.3
Herts/33	AY741404	II-IV	72.8	89.3	74.6	65.0	74.9	70.5	69.1	75.3	56.5
anhinga/U.S.(Fl)/44083/93	AY562986	II-V	72.0	85.7	74.6	63.4	72.1	69.5	68.6	74.9	56.5
pigeon/Yunnan/1111/2013	KM374056	II-VI	72.3	85.7	74.1	64.8	73.9	69.6	68.8	74.9	53.0
ZJ1	AF431744	II-VII	72.1	89.3	74.5	63.3	73.6	69.4	68.9	74.8	52.2
QH1	FJ751918	II-VIII	72.2	89.3	74.9	64.3	72.6	69.7	68.7	74.9	53.0
F48E8	FJ436302	II-IX	72.8	91.1	75.7	65.1	74.6	70.3	69.4	75.0	53.9

The predicted six genes and their encoded proteins are shown in Table 2. The lengths of 3’ leader and 5’ trailer were 55 and 114 nt respectively as reported for other NDV strains, and the 5’ untranslated regions (UTRs) of six genes were always longer than 3’ UTRs. The gene start (GS) sequence of each gene was ACGGGTAGAA, while the gene end (GE) sequence for NP, M, HN and L was TTAGAAAAAA, for that P gene was TAAGAAAAAA, and for that F gene was TTAAAAAAAA. The lengths of intergenic sequence (IGS) of P-M and M-F were 1 nt, while the IGS of NP-P, F-HN and HN-L were 2 nt, 31 nt and 48 nt, respectively.

**Table 2:** Genomic characteristics of NDV isolate GX1261.

Region	Gene sequence (nt)	3’ UTR (nt)	ORF (nt)	5’ UTR (nt)	Intergenic region (nt)	Nucleotide length (nt)	Amino acid length (aa)
Leader	1–55					55	
NP	56–1,801	66	122–1,591	210	2	1,746	489
P	1,804–3,266	83	1,887–3,086	180	1	1,463	399
M	3,268–4,508	34	3,302–4,396	112	1	1,241	364
F	4,510–6,301	46	4,556–6,217	84	31	1,792	553
HN	6,333–8,333	91	6,424–8,181	152	48	2,001	585
L	8,382–15,084	11	8,393–15,007	77		6,703	2,204
Trailer	15,085–15,198					114	

The cleavage site of F protein in GX1261 was ^112^ERQER/L^117^, which was typical of low virulence NDV. There were six potential glycosylation sites, Asn-X-Ser/Thr (N-X-S/T), in the F protein, which were highly conserved in most NDVs. Analysis of amino acids in the functional domain of the F protein showed GX1261 had 2 amino acid mutations in fusion peptide, 5 mutations in the heptad repeat region (HR), and 5 in the transmembrane domain, when compared with consensus amino acid sequences derived from NDV strains of different genotypes [19] and vaccine strains La Sota and V4 (Table 3). In the signal peptide, one amino acid difference at position 21 was identified between GX1261 (T) and other class I strains (S or I).

**Table 3:** Amino acid substitutions in the functional domains of the F protein.

Strains	Fusion peptide(117–141 aa)		HRa(143–185 aa)		HRb(268–299 aa)	HRc(471–500 aa)		Transmembrane domain(501–521 aa)				
	118	139	153	170	270	479	494	509	511	513	514	517
Consensus^a^	I	A	R	D	T	D	K	V	S	V	F	L
GX1261	V	S	K	S	S	G	R	T	A	I	C	V
Goose/Alaska/415/91	V	S	K	S	S	-	R	T	A	I	C	V
JS10	V	S	K	S	S	-	R	T	A	I	C	V
La Sota	-^b^	-	-	-	-	N	-	I	-	-	-	-
V4	-	S	-	N	-	-	-	-	-	-	-	-

^a^ The consensus amino acid sequence was derived from NDV strains of different genotypes

^b^ Same amino acid as that in the consensus amino acid sequence

The HN protein of GX1261 consisted of 585 amino acids (aa), which is the same as JS10, but different from Goose/Alaska/415/91 (616 aa). The sialic acid binding sites and cysteine residues in GX1261 were completely conserved as occurs with most NDVs. Four potential glycosylation sites at positions 119 (NAS), 341 (NDT), 433 (NKT), 481 (NHT), and 7 substitutions in the neutralizing epitopes were identified in the HN protein of GX1261 (Table 4).

**Table 4:** Amino acid substitutions in the neutralizing epitopes of the HN protein.

Strains	Neutralizing epitopes			
	263	332–333	346–353	513–521
Consensus^a^	N	GK	DEQDYQIR	RITRVSSSS
GX1261	Q	K333Q	E347D D349E I352V R353K	I514V
Goose/Alaska/415/91	Q	K333Q	E347D D349E I352V	I514V
JS10	Q	K333Q	E347D D349E I352V	I514V
La Sota	-^b^	-	-	-
V4	-	-	R353Q	-

^a^ The consensus amino acid sequence was derived from NDV strains of different genotypes

^b^ Same amino acid as that in the consensus amino acid sequence

### Phylogenetic analysis

**Fig 2:**
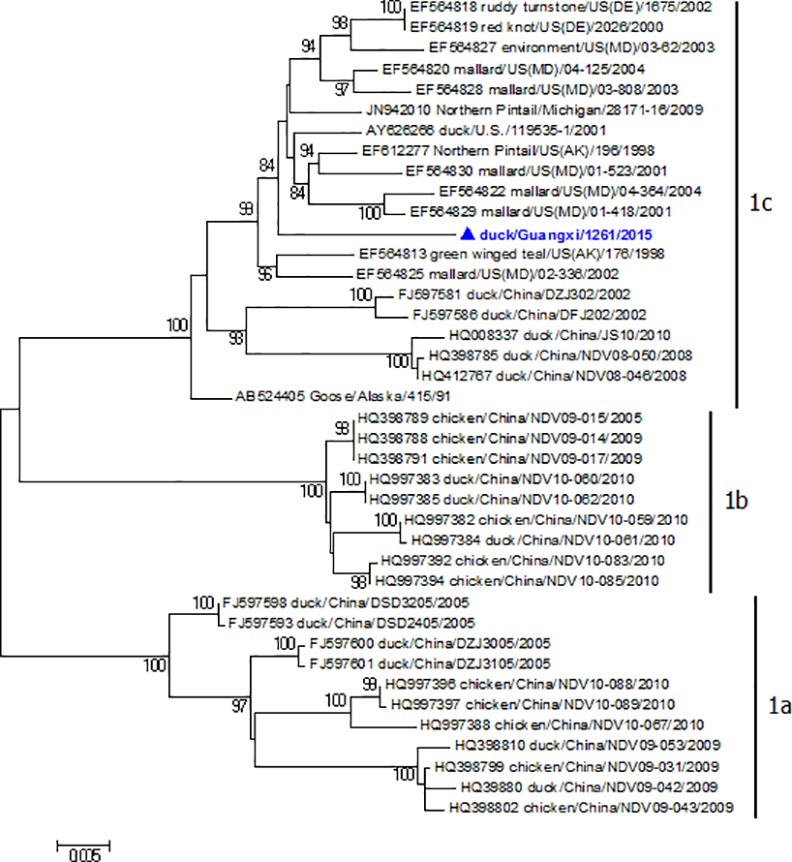
Phylogenetic analysis based on the F gene ORF of class I NDVs using the new classification system.

**Fig 3:**
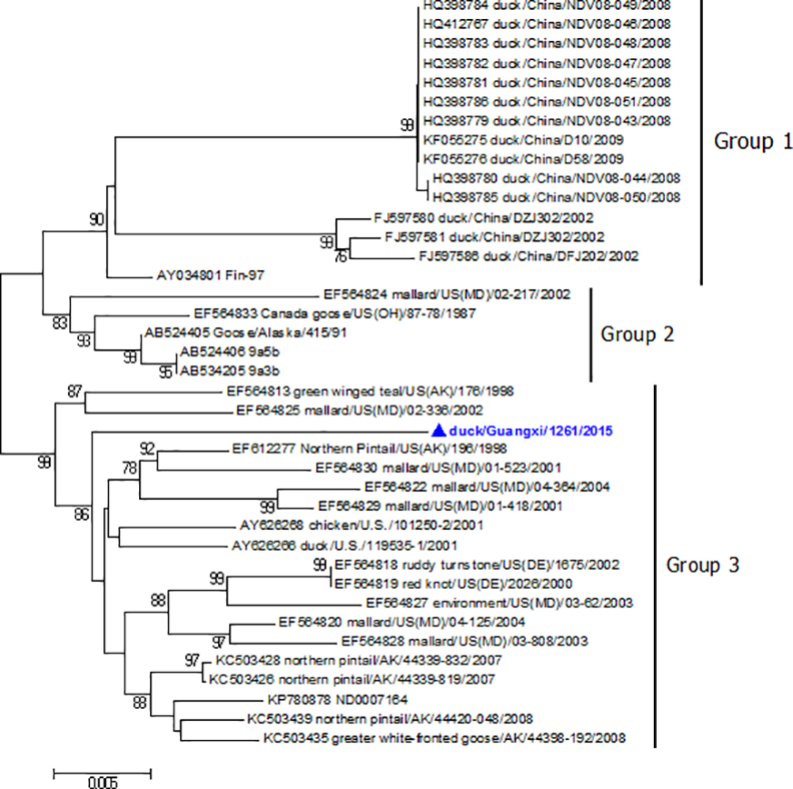
Phylogenetic analysis based on the F gene ORF of class I NDVs belonging to sub-genotype 1c.

Phylogenetic trees were constructed based on the complete genome or ORF of F gene. Phylogenetic analysis using the complete genome sequences revealed that GX1261 belonged to class I (S1 Fig). Sub-genotype analysis based on the F gene ORF using the new classification system proposed by Diel *et al* [8] showed GX1261 was clustered into sub-genotype 1c (Fig 2). Then, the sub-genotype 1c was further subdivided into 3 groups, and the result showed GX1261 belonged to group 3, which consisted of viruses isolated from waterfowl in the United States of America, while the other NDVs isolated in China all belonged to group 1 (Fig 3). The mean distance between groups 1 and 3 was 3.4%. These results of phylogenetic analysis indicated GX1261 was different from those class I NDVs circulating in China.

### Nucleotide sequence accession numbers

The complete genome sequence of the class I NDV isolate characterized in the study has been deposited in GenBank under the accession number KU748779.

## Discussion

Lentogenic NDVs belonging to class I and genotypes I and II of class II are commonly isolated from apparently healthy domestic poultry and wild birds, and numbers of class I viruses obtained from wild waterfowl and LBMs are increasing in these years [20,21]. In this study, one class I virus isolated from a symptomless duck in China was characterized genotypically. The role of class I NDV isolates with wide distribution in LBMs need to be evaluated in the evolution of NDV.

Based on the analysis of the F protein cleavage site, GX1261 was characterized as a low virulence strain. The complete genome and six gene sequences were compared with those of 12 representative viruses belonging to different genotypes in class I and class II. The identity of GX1261 genome sequence as compared with those of the reference strains varied from 97.6% (Goose/Alaska/415/91) to 72.0% (anhinga/U.S.(Fl)/44083/93), indicating the differences in genomic sequence between class I and class II were very distinct. Analysis of identity showed that GX1261 was most closely related to class I strain Goose/Alaska/415/91, which was isolated from North America. Since the two viruses were sampled from different places and in different years, it is highly likely that other similar viruses exist in the waterfowl in China or surrounding countries but as yet remain undetected or unidentified.

Several amino acid substitutions in the functional domains of F and HN proteins were identified by comparison of NDV sequences available in GenBank and GX1261 isolate. However, most of these amino acids were class I-specific, only two (21T, 479G) were different from class I strains. These were located in signal peptide and HRc region of the F protein, respectively. The signal peptide has an effect on the fusogenicity efficiency for the protein. As reported, the amino acid at position 27 located in the signal peptide could affect viral virulence in chicken [22]. Amino acid substitutions at fusion peptide and HR region, or replacement of transmembrane domain of NDV could affect the fusion activity of F protein [19]. In HN protein, when compared with the vaccine strains La Sota (genotype II in class II) and V4 (genotype I in class II), which are commonly used as live vaccines in China, GX1261 had seven amino acid differences in the neutralizing epitopes. Amino acid substitution in neutralizing epitopes was reported to play an important role in the formation of antigenic epitopes and could result in neutralizing escape variants [23,24,25]. Typically, NDVs contain six potential glycosylation sites at positions 119, 341, 433, 481, 508 and 501 [26], but GX1261 only had four potential glycosylation sites, unlike commonly used vaccine strains. Whether these amino acid substitutions in neutralizing epitopes and potential glycosylation sites of HN protein could change the antigenicity of GX1261 needs to be further studied.

Diversity of HN protein has been identified among class II NDV isolates. The size of HN protein (571, 577 or 616 aa) was related to the genotype of NDVs in class II [27]. Some studies showed that the length of HN protein might contribute to viral virulence [28]. In class I viruses, six different sizes (572, 574, 580, 581, 585, 616 aa) of HN proteins were identified based on analysis of 99 sequences obtained from GenBank, in which most viruses (73.7%) had HN protein of 616 aa, while only 18 viruses had the same HN size (585 aa) as GX1261. No relationship was identified between HN protein sizes and genotypes or sub-genotypes in class I NDVs.

Phylogenetic analysis of the F gene (1-1662nt) based on the new classification system proposed by Diel et al [8] showed that GX1261 belonged to sub-genotype 1c, and had higher similarity with the strains isolated from waterfowl in the United States of America before 2010 (group 3), while the other NDVs obtained from China all belonged to another group (group 1). Due to the lack of systemic surveillance in China in recent years, the generation of GX1261 and its epidemiological significance are not known. We believe that there may be some other similar class I viruses that exist in waterfowl in China, and so it is necessary to enhance the active surveillance of NDVs in waterfowl in case of emergence and spread of viruses.

In summary, this study described the whole genome characteristics of a class I NDV (GX1261) isolated from a LBM in China in 2015, in which several substitutions were observed in the functional domains of F and HN proteins when compared with the sequences of commonly used vaccine strains. Our study indicated that GX1261 could produce asymptomatic infection in ducks, and some other similar viruses may exist in waterfowl. Therefore, it is necessary to do more investigation to identify the source of the virus, and enhance active surveillance for NDV in waterfowl to assess the potential for virus shedding and transmission.

## Supporting Information

S1 Fig Phylogenetic analysis of class I isolate GX1261 based on the complete genome. (TIF)

S1 Table RT-PCR primers used for genome amplification. (DOCX)

S2 Table RT-PCR primers used to amplify 3’ leader and 5’ trailer. (DOCX)
