# Supplementary figures and images for: Genomic Characterizations of a Newcastle Disease Virus Isolated from Ducks in Live Bird Markets in China
Source: PLoS One. 2016 Jul 8;11(7):e0158771. doi: 10.1371/journal.pone.0158771 (PMC4938494; doi:10.1371/journal.pone.0158771)

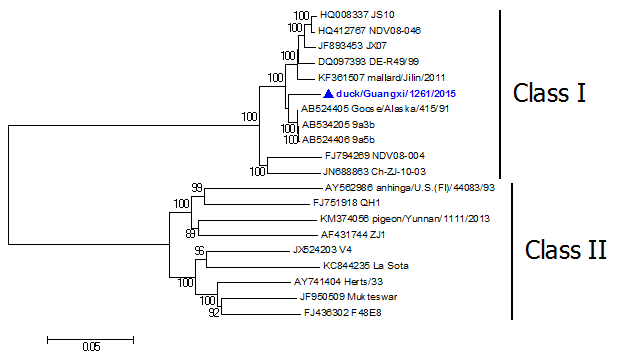

Supplement: S1 Fig — (TIF) [file pone.0158771.s001.tif]
