# Supplementary material for: Genomic Characterizations of a Newcastle Disease Virus Isolated from Ducks in Live Bird Markets in China
Source: PLoS One. 2016 Jul 8;11(7):e0158771. doi: 10.1371/journal.pone.0158771 (PMC4938494; doi:10.1371/journal.pone.0158771)
Supplement: S1 Table — (DOCX) [file pone.0158771.s002.docx]

S1 Table. RT-PCR primers used for genome amplification

| Name | Sequence (5’→3’) | Amplified product (bp) |
| --- | --- | --- |
| 1-F | AAGATGTCCTCCGTATTCG | 119-1760 |
| 1-R | GTGTGGTGATTGGTGTTG |  |
| 2-F | TGAGACCCAGTTTCTTGAT | 1450-3288 |
| 2-R | GGATGCTCCGATTCTACC |  |
| 3-F | GCCTTGCACTGAATGGAT | 3067-4695 |
| 3-R | TCTCCTGTGACTACTATTCCT |  |
| 4-F | ATGGCAGGCAGAATGGAT | 4544-6129 |
| 4-R | ATATAGCAGGCGAGGACTAA |  |
| 5-F | AGTTAATGTGAGGCTTACCA | 6025-7739 |
| 5-R | TGGAGAGTCGCAGTCTTA |  |
| 6-F | GACTTCTAATACCATCACTCTC | 7593-9207 |
| 6-R | TCCACCAGCCTCAAGATA |  |
| 7-F | TGTGTTGATGTATGCGGATA | 9091-10872 |
| 7-R | TTCTTGAGGACCTGACTGA |  |
| 8-F | ATATTGTCAGTGCGAGAG | 10518-12428 |
| 8-R | TGCTATGGAGGTGGAGAG |  |
| 9-F | AGGCTGTTCACTGAGGAG | 12287-14009 |
| 9-R | GGTAAGGTACTACGGATGTAAT |  |
| 10-F | CTCTGTATCTCGCTGAAGG | 13713-15135 |
| 10-R | TGACACTATGACTCGGTTG |  |
