# Supplementary material for: Genomic Characterizations of a Newcastle Disease Virus Isolated from Ducks in Live Bird Markets in China
Source: PLoS One. 2016 Jul 8;11(7):e0158771. doi: 10.1371/journal.pone.0158771 (PMC4938494; doi:10.1371/journal.pone.0158771)
Supplement: S2 Table — (DOCX) [file pone.0158771.s003.docx]

S2 Table. RT-PCR primers used to amplify 3’ leader and 5’ trailer

| Name | Sequence (5’→3’) | Position (nt) |
| --- | --- | --- |
| 3’ outer | CGAGTGAGAGCACAGTAGAGAT | 343-364 |
| 3’ inner | GCAATCCGAAGGCAGAATAC | 278-297 |
| 5’ outer | TAACACGAGCACAACAAAA | 14928-14946 |
| 5’ inner | GGGAACGCTGTCAAAGGATA | 14966-14985 |
